# Cloning and Characterization of *ThSHR*s and *ThSCR* Transcription Factors in *Taxodium* Hybrid ‘Zhongshanshan 406’

**DOI:** 10.3390/genes8070185

**Published:** 2017-07-20

**Authors:** Zhiquan Wang, Yunlong Yin, Jianfeng Hua, Wencai Fan, Chaoguang Yu, Lei Xuan, Fangyuan Yu

**Affiliations:** 1 Collaborative Innovation Center of Sustainable Forestry in Southern China, College of Forest Sciences, Nanjing Forestry University, Nanjing 210037, China; zhiquanjiejie@163.com (Z.W.); yinyl066@sina.com (Y.Y.); 2 Institute of Botany, Jiangsu Province and Chinese Academy of Sciences, Nanjing 210014, China; jfhua2009@gmail.com (J.H.); fanfan461@sina.com (W.F.); yuchaoguang888@sina.com (C.Y.)

**Keywords:** SHORT-ROOT, SCARECROW, adventitious root development, *Taxodium*

## Abstract

Among the GRAS family of transcription factors, SHORT ROOT (SHR) and SCARECROW (SCR) are key regulators of the formation of root tissues. In this study, we isolated and characterized two genes encoding SHR proteins and one gene encoding an SCR protein: *ThSHR1* (Accession Number MF045148), *ThSHR2* (Accession Number MF045149) and *ThSCR* (Accession Number MF045152) in the adventitious roots of *Taxodium* hybrid ‘Zhongshanshan’. Gene structure analysis indicated that *ThSHR1*, *ThSHR2* and *ThSCR* are all intron free. Multiple protein sequence alignments showed that each of the corresponding proteins, ThSHR1, ThSHR2 and ThSCR, contained five well-conserved domains: leucine heptad repeat I (LHRI), the VHIID motif, leucine heptad repeat II (LHR II), the PFYRE motif, and the SAW motif. The phylogenetic analysis indicated that ThSCR was positioned in the SCR clade with the SCR proteins from eight other species, while ThSHR1 and ThSHR2 were positioned in the SHR clade with the SHR proteins from six other species. Temporal expression patterns of these genes were profiled during the process of adventitious root development on stem cuttings. Whereas expression of both *ThSHR2* and *ThSCR* increased up to primary root formation before declining, that of *ThSHR1* increased steadily throughout adventitious root formation. Subcellular localization studies in transgenic poplar protoplasts revealed that ThSHR1, ThSHR2 and ThSCR were localized in the nucleus. Collectively, these results suggest that the three genes encode *Taxodium* GRAS family transcription factors, and the findings contribute to improving our understanding of the expression and function of SHR and SCR during adventitious root production, which may then be manipulated to achieve high rates of asexual propagation of valuable tree species.

## 1. Introduction

Rapid increases in atmospheric CO_2_ concentration have resulted in climate change, such as global warming [1]. Afforestation and reforestation are recognized as economically efficient ways of ameliorating the problems associated with elevated atmospheric CO_2_ by increasing the extent of the forest carbon sink. The most widely-used method of vegetative propagation in forestry is stem cuttings from elite genotypes. The process of adventitious root formation and development, which is crucial to stem cutting, can be distinguished into three phases, induction, initiation and expression, according to previous reports [2]. However, for many ecologically and economically important tree species the inability of stem cuttings to form adventitious roots hinders the development of large-scale plantations [3]. In response to this problem, a number of studies on important tree species, investigating the process of adventitious root formation at the physiological, anatomical, biochemical and molecular levels, have been undertaken to overcome these problems [4,5]. Identification and characterization of the genes controlling adventitious root formation are essential to understanding and potentially manipulating this phenomenon, in order to propagate more tree species from cuttings.

The primary root and lateral roots build the adventitious root system jointly. SHORT ROOT (SHR) and SCARECROW (SCR) are part of the GRAS family of transcription factors, named after the first three members: GIBBERELLIC-ACID INSENSITIVE (GAI), REPRESSOR of GAI (RGA) and SCARECROW (SCR) and are key regulators of primary root stem cell definition and maintenance, and of radial patterning [6,7,8]. During, *Arabidopsis* development the intercellular movement of SHR and the subsequent interaction with its downstream target gene *SCR* control root patterning and cell fate specification [9]. In the *Arabidopsis* root, *SHR* is expressed in the stele, then SHR moves into the adjacent cell layer to upregulate *SCR* transcription and control endodermis specification [10,11,12]. In comparison, *SCR* is expressed in the root cortex-endodermis initial cell and the endodermis of *Arabidopsis* root. The SCR protein, in turn, binds to its own promoter in the presence of SHR and JACKDAW (JKD)/IDD10, and they together regulate quiescent center markers and microRNAs involved in primary root vascular differentiation [13,14,15]. Loss-of-function mutations in *SHR* or *SCR* result in abnormal root development, indicating that SHR and SCR are essential for regulation of the asymmetric cell divisions which generate the root ground tissue; *shr* mutant roots lack the endodermis layer while the root ground tissue of *scr* mutants has a mixed cortex/endodermis identity [5,16,17].

Despite the SHR/SCR network in the root system being investigated in detail in herbaceous model plants [12,18], there is no report of the isolation and characterization of the *SHR* and *SCR* genes in the adventitious roots of *Taxodium* hybrid ‘Zhongshanshan’. *T.* ‘Zhongshanshan’ are interspecies hybrid clones generated from three *Taxodium* species, *Taxodium mucronatum*, *Taxodium distichum*, and *Taxodium ascendens*, and have been widely used as timber trees in river network areas, as windbreak trees in coastal areas, and as landscape trees in urban areas because of their extreme tolerance to a number of abiotic stresses, such as high salinity and waterlogging [19,20]. In this study, we identified two genes encoding SHR proteins (*ThSHR1* and *ThSHR2*) and one gene encoding an SCR protein (*ThSCR*) from *Taxodium* hybrid ‘Zhongshanshan 406’, and characterized the genes with respect to their gene structures, sequence similarities, expression patterns and subcellular localizations.

## 2. Materials and Methods

### 2.1. Plant Growth Conditions

Softwood cuttings of *Taxodium* hybrid ‘Zhongshanshan 406’ (*T. mucronatum* ♀ × *T. distichum* ♂) were collected from the Institute of Botany, Jiangsu Province & Chinese Academy of Sciences, Nanjing, China. In July 2016, about 600 one-year old healthy softwood cuttings were selected for the experiment from the tops of mother seedlings less than 8-years old. Each cutting was then cut into a length of about 15 cm, and one-third to one-half of the leaves on each cutting were removed to prevent excessive moisture loss. Then, 1000 mg/L carbendazim was used to thoroughly spray the seedbed (containing moistened perlite and peat soil with organic matter, 2:1) in a ventilated greenhouse under normal growth conditions (approximately 30 °C), in a photoperiod of 10/14 h of light/dark, to prevent mildew and other fungal attacks. After seedbed treatment, the cuttings were planted individually in the seedbed. The relative humidity was set at 70–80%.

**Figure 1:**
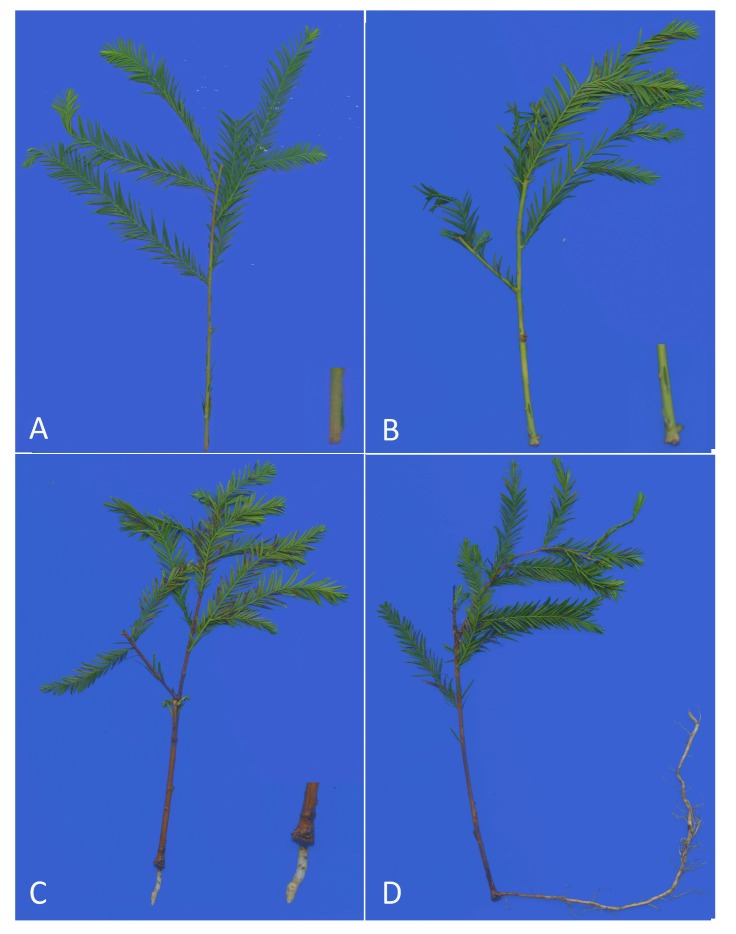
Adventitious root formation in *Taxodium* hybrid ‘Zhongshanshan 406’ at different developmental time points: (**A**) the control time point (S0); (**B**) the initial formation of callus (S1); (**C**) the formation of primary root (S2); and (**D**) lateral root systems (S3).

Based on the three phases of adventitious root formation and development, induction, initiation and expression, samples of cuttings were taken at two significant time points based on apparent morphological changes. The first time point (S1) was the initial formation of callus, the second (S2) was the time point when the primary root formed. And another time point, the lateral root formed period(S3), was added. The control time point (S0) was taken at 0 day, with the cuttings stored immediately after excision. The basal stem tissue (0.2–0.5 cm) (at S0 and S1) and the root tissue (at S2 and S3) of the cuttings were sampled for analysis at these four specific time points (Figure 1), at which the tissues were frozen immediately in liquid nitrogen and stored at −80 °C until needed for extraction of RNA and DNA. Three independent biological replicates, each consisting of 20 randomly-selected stem cuttings, were taken at each time point.

MS agar medium was selected to cultivate the elite clone of hybrid poplar (*Populus deltoides* × *Populus euramericana* cv. ‘Nanlin895’) for protoplast transfection under light intensity of 350 μmol·m^−2^·s^−1^ in the photoperiod of 16/8 h of day/night temperatures of 25/18 °C. Newly-expanded young leaves of 6-week-old plants were used for protoplast isolation.

### 2.2. Gene Cloning

Total RNA was extracted using the RNAprep Pure Plant Kit (Polysaccharides- & Polyphenolics-rich) (Tiangen, Beijing, China) and was treated with RNase-free DNase I (Qiagen, Dusseldorf, Germany). RNA concentration was measured with a spectrophotometer (NanoDrop2000, Thermo Scientific, Waltham, MA, USA) at 260 and 280 nm, and samples with the 260/280 nm ratio within the range 1.80–2.20 were used. RNA integrity was also checked using 2% agarose gels. The DNase-treated RNA of adventitious roots (S2 and S3) was used for rapid amplification of cDNA ends (RACE). Based on sequences provided from the previously-obtained transcriptomics data (unpublished data), fragment sequences, from the middle region, were PCR-amplified and sequenced [19,20]. Then, based on the fragment sequences, the nested primers were designed using the Oligo 6.0 software (Molecular Biology Insights, Colorado Springs, CO, USA) to amplify the full-length sequences with the 3-Full RACE Core Set Kit (TaKaRa, Otsu, Japan) and SMARTer RACE 5′/3′Kit (Clontech, Palo Alto, CA, USA), according to the manufacturer’ s instructions. The PCR products were recovered and cloned into pMD19-T vectors (TaKaRa), finally transformed into competent cells of the *Escherichia coli* strain DH5α, and sent to the sequencing facility. By comparing and aligning the sequences of 3′-RACE and 5′-RACE and the middle region products, the full-length cDNA sequences of the two *ThSHR*s and of *ThSCR* were obtained. The predicted open reading frames (ORFs) were then amplified by PCR, and were verified by sequencing. Genomic DNA was extracted using a Plant Genomic DNA Extraction Kit (BioTeke, Beijing, China). Genomic DNA sequences of the ORFs of the above genes were amplified from RNase-treated DNA using the same primers, and were verified by sequencing. The sequences of the primers are listed in Table 1.

### 2.3. Sequence Analysis

Online BLAST software was used to analyze the DNA and protein sequences (https://blast.ncbi.nlm.nih.gov/Blast.cgi). ORFs were predicted with the ORFfinder program (https://www.ncbi.nlm.nih.gov/orffinder/). Gene predictions were performed using the FGENESH program(http://linux1.softberry.com/berry.phtml?topic=fgenesh&group=programs&subgroup=gfind). The theoretical isoelectric point (pI), molecular weight (MW) and amino acid composition of the proteins were predicted and calculated using Expasy Protparam (http://web.expasy.org/protparam/). Protein transmembrane structures, protein domain and signal peptide cleavage site analyses were performed using the TMHMM (http://www.cbs.dtu.dk/services/TMHMM/), PROSITE (http://prosite.expasy.org/) and SignalP online tools (http://www.cbs.dtu.dk/services/SignalP/), respectively. Secondary structures of deduced amino acid sequences were predicted by the GOR IV secondary structure prediction method (https://npsa-prabi.ibcp.fr/cgi-bin/npsa_automat.pl?page=npsa_gor4.html). The deduced protein sequences of ThSHR1, ThSHR2 and ThSCR were aligned with other plant SHR and SCR sequences using the BioEdit 2.6 software (http://www.mbio.ncsu.edu/BioEdit/bioedit.html). The putative nuclear localization signals were predicted with NLStradamus (http://www.moseslab.csb.utoronto.ca/NLStradamus/). The phylogenetic tree was constructed using MEGA 5.0 with Maximum Likelihood method and 1000 bootstraps [21].

### 2.4. Real Time PCR Analysis of ThSHRs and ThSCR

The complementary DNA (cDNA) was synthesized for real-time PCR based on 1 μg DNase-treated RNA using PrineScript@RTase (200 U) system (TaKaRa), according to the manufacturer’s instructions. Primers for the reference gene adenine phosphoribosyl transferase (*APRT*) and the three target genes were designed using the Oligo 6.0 software with the following parameters: melting temperatures of 58–62 °C, primer lengths of 19–25 bp, GC content of 40–60% and amplicon lengths of 80–210 bp (Table 1) [22]. Real-time PCR was conducted in 96-well plates and performed on the Analitik Jena qTOWER2.2 PCR System (Biometra, Gottingen, Germany) using the following cycling conditions: 50 °C for 2 min, 95 °C for 10 min, and 40 cycles of 95 °C for 15 s, 60 °C for 1 min followed by a melting curve analysis by heating the PCR products from 60 to 95 °C. Each reaction mix contained 2 μL cDNA of previously diluted cDNA (1:3), 10 μL FastStart Universal SYBR Green Master (ROX, Roche Applied Science, Mannheim, Germany), 6.8 μL RNase-free water, and 6 pmol of each primer, for a final volume of 20 μL. A no-template control was also included in each run for each gene. Each real-time PCR analysis was performed in triplicate. The calculations of relative expression levels between the target and reference genes were performed using the delta-Ct method [23,24].

### 2.5. Protoplast Transfection

In this study, plasmids were constructed by Gateway Technology (Invitrogen, Leek, The Netherlands), following the manufacturer’s protocol. First, the *ThSHR* (or *ThSCR*) coding regions (without the stop codon) were cloned into the entry vector, pCR8/GW/TOPO (Invitrogen), with a simple TOPO cloning reaction. In order to perform the subcellular localization of tagged proteins, an LR clonase enzyme mix (Invitrogen) was used to transfer the insert from the entry vector into its destination vector, p2GWF7, with a green fluorescent protein (GFP) tag positioned at the C-terminal of the insert. The GFP fusion vectors (35S::*ThSHR1*-GFP,35S::*ThSHR2*-GFP, and 35S::*ThSCR*-GFP) generated were high-copy vectors, driven by the promoter of double 35S cauliflower mosaic virus with ampicillin as the selectable bacterial marker. Protoplast isolation and polyethylene glycol-mediated transfection were performed using the method described by [25]. A BX51 fluorescence microscope (Olympus America, Center Valley, PA), equipped with 450–490 nm/ 520–560 nm and 540–580 nm/600–660 nm (excitation/emission) filters, was used to capture images.

## 3. Results

### 3.1. Molecular Cloning and Sequence Analysisof ThSHR and ThSCR Genes

Three target genes, termed *ThSHR1*, *ThSHR2* and *ThSCR*, were successfully isolated and identified by 3′-RACE and 5′-RACE procedures. Comparisons of genomic and cDNA sequences showed that *ThSHR1*, *ThSHR2* and *ThSCR* were all intron-free. The full-length sequence of *ThSHR1* cDNA was 2069 bp, containing an ORF of 1455 bp, flanked by 332 bp of 5′-untranslated region (UTR) and a 282 bp 3′-UTR; *ThSHR2* was 1515 bp with an ORF of 1290 bp, flanked by 52 and 173 bp 5′- and 3′-UTRs, respectively; *ThSCR* was 1909 bp with an ORF of 1596 bp, flanked by 220 and 93 bp 5′- and 3′-UTRs, respectively (Table 2).

Genes *ThSHR1*, *ThSHR2* and *ThSCR* encode polypeptides of 484, 429 and 531 amino acid residues, respectively. The GOR IV secondary structure prediction method was used to predict the secondary structures of these three proteins [26]. TheThSHR1, ThSHR2 and ThSCR proteins contained 41.32%, 32.87% and 38.61% of alpha helices (Hh), respectively; and correspondingly 14.46%, 19.11% and 17.33% of extended strands (Ee), respectively; and 44.21%, 48.02%and 44.07% of random coils (Cc), respectively; interestingly, the three proteins all contained zero beta turns (Tt) (Table 2). The Prosite reports suggested that the three proteins had a typical GRAS domain: amino acid residues 89–462 for ThSHR1 (score 51.621), 38–407 for ThSHR2 (score 50.775) and 138–505 for ThSCR (score 58.917) [27].

**Figure 2:**
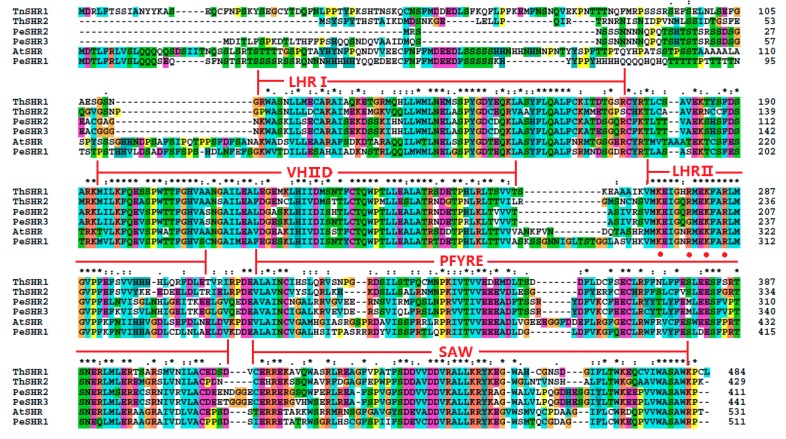
Alignment of the amino acid sequences of ThSHR1 and ThSHR2 with those of SHORT ROOT (SHR) of both *Arabidopsis* and *Populus*, using the ClustalW program. The highly-conserved region of the GRAS proteins can be divided into five recognizable motifs: the leucine heptad repeat I (LHR1), the VHIID motif, the leucine heptad repeat II (LHRII), the PFYRE motif and the SAW motif, as indicated above the sequence alignment. Amino acid position numbers are indicated to the right of the sequence. The putative nuclear localization signals were labeled with red points. The same below.

**Figure 3:**
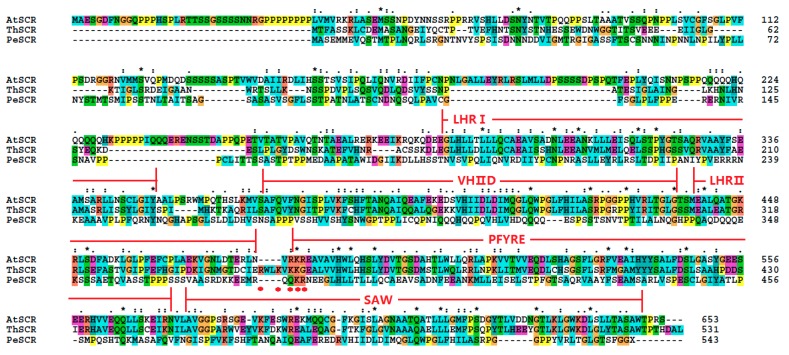
Alignment of the amino acid sequences of ThSCR with those of SCARECROW (SCR) of both *Arabidopsis* and *Populus*.

The protein sequences of *ThSHR1*, *ThSHR2* and *ThSCR* were compared with *Arabidopsis* protein sequences in TAIR (AtSHR, At4g37650.1; AtSCR, At3g54220.1) and *Populus* protein sequences based on previous studies [28,29,30,31]. The result indicated that the two SHR proteins and one SCR protein belonged to the plant-specific GRAS family of transcription factors and exhibited considerable sequence similarity to one other throughout their carboxyl termini, including leucine heptad repeat I (LHRI), the VHIID motif, leucine heptad repeat II (LHRII), the PFYRE motif and the SAW motif (Figure 2 and Figure 3). The putative nuclear localization signals were marked with red points in Figure 2 and Figure 3.

**Figure 4:**
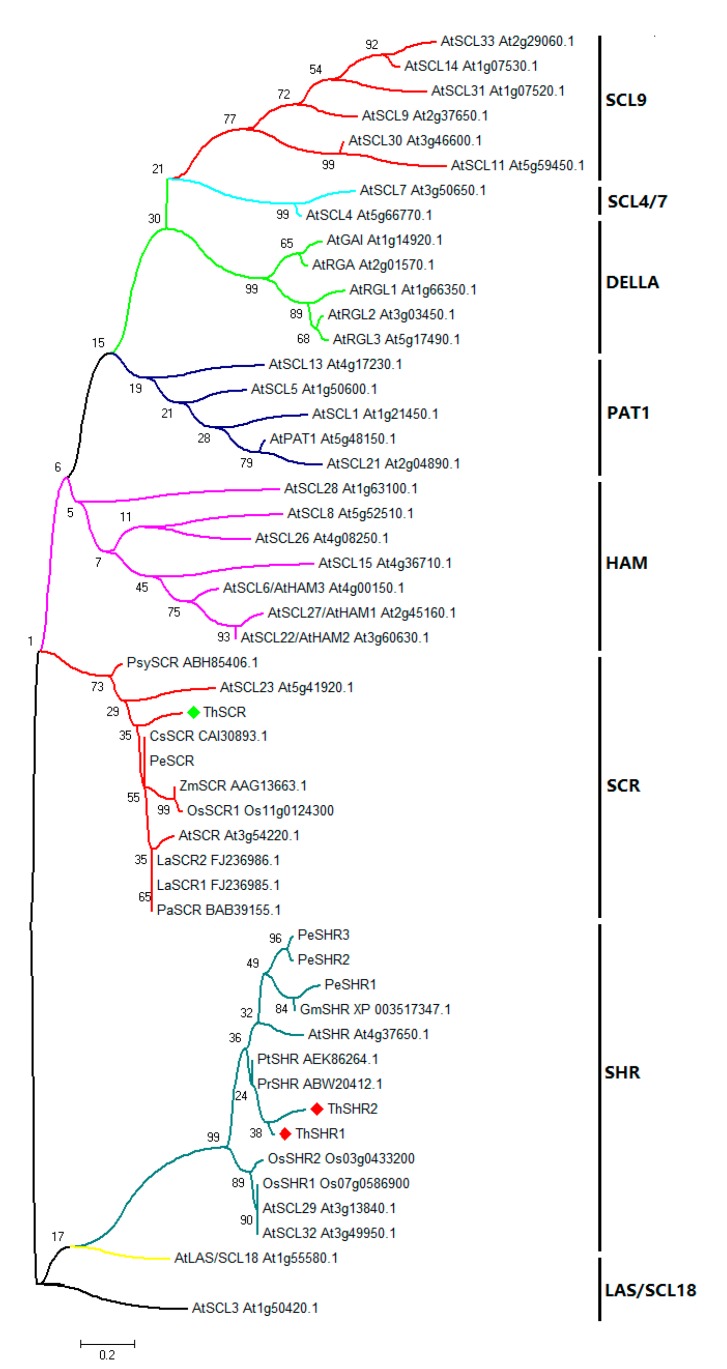
Phylogenetic analysis of the proteins of the GRAS gene family. The phylogenetic tree was constructed using MEGA 5.0 with the maximum likelihood method using 1000 replicate bootstrap tests. Numbers near to the nodes indicate bootstrap values obtained from 1000 replications. The 51 proteins were clustered into eight distinct groups: SCL9, PAT1, LAS/SCL18, SCL4/7, SHR, SCR, HAM and DELLA, as indicated. At, *Arabidopsis thaliana*; Os, *Oryza sativa*; Gm, *Glycine max*; Pe, *Populus*; Pr, *Pinus radiata*; Pt, *Pinus taeda*; La, *Lupinus albus*; Ps, *Pisum sativum*; Cs, *Cucumis sativa*; Zm, *Zea mays*; Psy, *Pinus sylvestris*.

To understand the evolutionary relationship of GRAS proteins from different species, the amino acid sequences of ThSHR1, ThSHR2 and ThSCR (GRAS domains) were aligned with those of 48 full-length GRAS proteins, and an un-rooted neighbor-joining (NJ) phylogenetic tree was constructed using MEGA 5.0 software and the maximum likelihood method with 1000 replicate bootstrap testing. The GRAS protein family can be divided into several clades, which have been designated after one of their members or their common features [32]. In this study, the un-rooted NJ tree based on multiple sequence alignments showed that the 51 protein domains were clustered into eight distinct groups (Figure 4). The result showed that ThSHR1 and ThSHR2, most closely related to the conifer proteins PrSHR (from *Pinus radiata*) and PtSHR (from *Pinus taeda*), were positioned in the SHR clade; ThSCR was positioned in the SCR clade, and had an identity of 52% with AtSCL23 (Figure 4).

### 3.2. Expression of ThSHR and ThSCR

**Figure 5:**
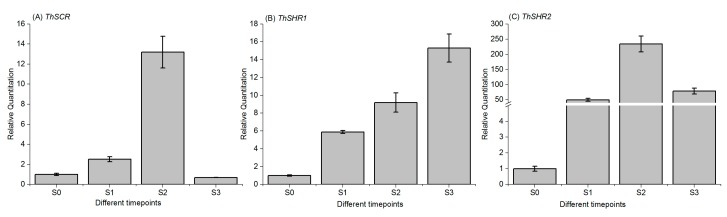
Temporal expression patterns of *ThSHR1*, *ThSHR2* and *ThSCR* by real-time PCR. S0, S1, S2 and S3 are the tissues at 0 day, initial formation of callus, primary root and the lateral roots, respectively. For real-time PCR, adenine phosphoribosyl transferase (*APRT)* was used as the internal control, and the relative transcript levels were calculated using the comparative delta-Ct method. Error bars represent the standard deviation for each sample, based on three biological replicates (the averages of technical replicates).

To analyze the expression patterns of the *ThSHR1*, *ThSHR2* and *ThSCR* genes in *T*. ‘Zhongshanshan 406’, we measured their transcript levels by real-time PCR at different developmental time points: the control time point (S0), the initial formation of callus(S1), the formation of primary root(S2) and lateral root systems(S3) (Figure 1). The gene expression patterns of *ThSHR2* and *ThSCR* were similar over the period of adventitious root formation, with an apparent increase-decrease trend over time. The maximum expression of both genes occurred at S2, with the minimum expression at either S3 (*ThSCR*) or S0 (*ThSHR2*). In contrast with these two genes, the expression pattern of *ThSHR1* showed a trend of increasing expression throughout the process of adventitious root formation (Figure 5).

### 3.3. Subcellular Localization of ThSHR and ThSCR Proteins

**Figure 6:**
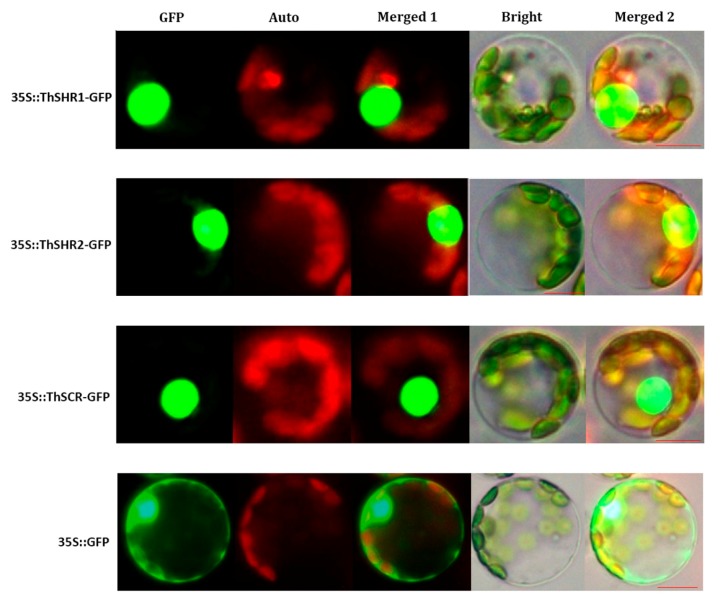
Subcellular localization of ThSHR1, ThSHR2 and ThSCR. Green fluorescent protein (GFP), chlorophyll auto fluorescence (Auto), merged1, bright and merged 2 images are shown. Scale bar: 5μm. The 35::GFP fusion was used as the positive protein control, and was detected in the nucleus and cytoplasm of *Populus* protoplasts.

In order to probe the subcellular localizations of the ThSHR1, ThSHR2 and ThSCR proteins, the GFP-fusion vectors (35S::ThSHR1-GFP, 35S::ThSHR2-GFP and 35S::ThSCR-GFP) were transformed into *Populus* protoplasts under the control of the double 35S cauliflower mosaic virus promoter. The cellular localization of the fusion protein was observed using confocal microscopy. All three proteins were located in the nucleus. As a positive control, the 35::GFP fusion protein was detected in the nucleus and cytoplasm of *Populus* protoplasts (Figure 6).

## 4. Discussion

Transcription factors (TFs), also known as trans-acting factors, are located in the nuclei and exhibit a special interaction with cis-regulatory elements in the gene promoter regions. As a result, expression of a set of downstream functionally-related target genes are regulated in a spatio-temporal specific manner [33]. Hence, TFs are core components of gene regulation networks [33,34]. SHR and SCR belong to the plant-specific GRAS TF family [32,35,36]. It has been reported that SHR and SCR play key roles in formation of root ground tissue [6,37]. They are essential to regulate the asymmetric cell divisions, and thereby to generate the root ground tissue [38,39,40]. To date, a number of SHR and SCR genes involved in seedling root system have been studied systematically [9,12,33]. However, studies in SHR and SCR genes involving adventitious roots were few and far between.

In this study, two ThSHR and one ThSCR genes, which may be involved in adventitious root development of *Taxodium* hybrid ‘Zhongshanshan’, were isolated and characterized in the adventitious roots. The gene structure analysis indicated that these three genes were all intron-free. Similar results were obtained in *Populus* [31]. The Prosite reports have suggested that the three proteins had a typical GRAS domain, thereby confirming that the three proteins belong to the plant-specific GRAS family of TFs [35]. The result of multiple sequence alignments indicated thatThSHR1, ThSHR2 and ThSCR showed considerable protein sequence similarity with other SHRs and SCRs of *Arabidopsis* and *Populus* throughout their carboxyl termini, including the LHRI, VHIID, LHRII, PFYRE and SAW motifs. On the other hand, the three proteins showed variation in the length and amino acid sequence at the amino-termini. Such variation may reflect the ability of the proteins to bind to different promoter nucleotide sequences and accessory proteins [32]. In the previous study, Pysh hypothesized that the area encompassing the two LRRs and the VHIID motif may act as a DNA binding domain analogous to the bZIP protein-DNA interaction domain, with the LRRs mediating protein-protein interactions, and the VHIID motif mediating DNA-protein interactions [41]. So, we also hypothesized that the area encompassing the LHRI and the VHIID motif may act as a DNA binding domain analogous.

Meanwhile, the phylogenetic analysis of plant GRAS proteins indicates thatThSHR1, ThSHR2 and ThSCR showed highly similarity to other GRAS proteins. ThSCR was positioned in the SCR clade with SCR proteins from eight other species, including *A. thaliana*, *O. sativa*, *Z. mays*, *Lupinus albus*, *Cucumis sativa*, *Populus*, *Pisum sativum* and *Pinus sylvestris*. ThSHR1 and ThSHR2 were positioned in the SHR clade with SHR proteins from six other species. Interestingly, the two ThSHR proteins were most closely related to two Pinus species, also conifers like *Taxodium*. On the whole, the results of this study are consistent with previous reports [32]. Consistent with their role as transcriptional regulators, the ThSHR1-GFP, ThSHR2-GFP and ThSCR-GFP fusion proteins were located in the nucleus, similar to the situation in *Populus* cells [31]. Combined together, these features provide strong evidence that the three genes isolated here encode *Taxodium* GRAS family transcription factors. The difference of localization of SHR of *Taxodium* to that of *Arabidopsis* maybe due to species difference, and this could be further investigated in future studies in this area [42,43].

In studying the expression patterns, we measured their transcript levels by real-time PCR at different developmental time points: the control time point (S0), the initial formation of callus (S1), the formation of primary root (S2) and lateral root systems (S3) (Figure 1). In contrast to previous studies, we added a stage after expression (lateral root formed period (S3)) to explore the subsequent expression of genes [2]. Expression patterns of these three genes showed that expression of the genes ThSHR2 and ThSCR was highest at the period of S2, then began to decline, while that of gene ThSHR1 exhibited steady increases in expression throughout the process of adventitious root formation and development. The expression pattern of ThSHR1 is similar to the previous reports, while ThSHR2 and ThSCR are not exactly consistent with previous studies [31]. The possible reasons are differences in design and differences in species. This result revealed that the *ThSHR1*, *ThSHR2* and *ThSCR* genes might be involved in the formation and development of adventitious roots. This finding contributes to improving our understanding of how genes *SHR* and *SCR* function in the development of adventitious root formation, and could result in advances in asexual propagation of normally recalcitrant species, such as the conifers. Future research is necessary to verify the roles of ThSHR and ThSCR by in situ hybridization, and their functions by genetic transformation. Elucidating whether these proteins interact with each other is also important.

## Figures and Tables

**Table 1:** Primer sequences of the genes for rapid amplification of cDNA ends (RACE), full-length open reading frames (ORFs) and real-time PCR (qRT-PCR).

Primer-ID	Forward PCR Primer (5′-3′)	Reverse PCR Primer (5′-3′)
ThSHR1_3OUTER	TGTGTGAGCGCCGTGAGAAGGCTGTGCA	TACCGTCGTTCCACTAGTGATTT
ThSHR1_3INNER	TTTTGCAGTTTTTTGTAAGTCCTG	CGCGGATCCTCCACTAGTGATTTCACTATAGG
ThSHR1_5OUTER	CTAATACGACTCACTATAGGGCAAGCAGTGGTATCAACGCAGAGT	ATCCATAGAAGATGCTGCATTCGC
ThSHR1_5INNER	CTAATACGACTCACTATAGGGC	GTTGGTGGTGGTATTGGGCTTCTC
ThSHR1_ORF	ATGGATAGATTGTTTACCTCCAGCATA	TTACAAGCAAGGCTTCCAGGCGG
ThSHR1_qRT-PCR	TACCCGCGACCTTCAGTGAC	CAGGCGGAAGCCCAAATGAC
ThSHR2_3OUTER	TTCTGTTATGAAGGAAATAGGGCA	TACCGTCGTTCCACTAGTGATTT
ThSHR2_3INNER	TCAGCCTGTGTTTCGTTTCGCTTGA	CGCGGATCCTCCACTAGTGATTTCACTATAGG
ThSHR2_5OUTER	CTAATACGACTCACTATAGGGCAAGCAGTGGTATCAACGCAGAGT	CTGAACTCAAACGGCACTCCCATAA
ThSHR2_5INNER	CTAATACGACTCACTATAGGGC	AGGGACTAGCCTCCTGGAATTTCAG
ThSHR2_ORF	ATGTCATATAGTTTTTATACCCATTC	CTATTTAGGTTTCCAGGCAGAAG
ThSHR2_qRT-PCR	ACTCAGTGGCCCATGCTGTT	AACGGCACTCCCATAAGCCT
ThSCR_3OUTER	ATCCTGATGACAGCATAGAAAGACA	TACCGTCGTTCCACTAGTGATTT
ThSCR_3INNER	TGGAACTCTCAAACTTGGCTGGAAAGAC	CGCGGATCCTCCACTAGTGATTTCACTATAGG
ThSCR_5OUTER	CTAATACGACTCACTATAGGGCAAGCAGTGGTATCAACGCAGAGT	ACATTGGAGTAGAAGATCCAGAAGG
ThSCR_5INNER	CTAATACGACTCACTATAGGGC	TCCAGTTTGCAGCTCCAATTTCATC
ThSCR_ORF	ATGACATTTGCTTCTTCTAAATTGTGTG	TTACAGTGCATCATGGGTGGGTGTC
ThSCR_qRT-PCR	ATGGCCAGGGCTATTCCACA	CTGCCTGTTGCCTCCAATGC
APRT_qRT-PCR	TCCACAGGTTCTTGAATCGCT	TGACTTGAGCCTCATTCGCTC

**Table 2:** Features of the *ThSHR1*, *ThSHR2* and *ThSCR.*

Gene_ID	Full-Length cDNA(bp)	5′-UTR (bp)	3′-UTR (bp)	ORF (bp)	Predicted Peptide			Secondary Structure Prediction			
					MW (kDa)	PI	GRAVY	Hh (%)	Ee (%)	Tt (%)	Cc (%)
***ThSHR1***	2069	332	282	1455	55.52	5.58	−0.383	41.32	14.46	0.00	44.21
***ThSHR2***	1515	52	173	1290	48.80	5.26	−0.256	32.87	19.11	0.00	48.02
***ThSCR***	1909	220	93	1596	59.12	5.43	−0.224	38.61	17.33	0.00	44.07

UTR: untranslated region; ORF: open reading frame; MW: molecular weight; PI: isoelectric point; GRAVY: grand average of hydropathicity; Hh: alpha helices; Ee: extended strands; Tt: beta turns; Cc: random coils.
